# MLPA as a complementary tool for diagnosis of chromosome 21 aberrations in childhood BCP-ALL

**DOI:** 10.1007/s13353-019-00509-8

**Published:** 2019-08-27

**Authors:** Ewa Wrona, Marcin Braun, Agata Pastorczak, Joanna Taha, Monika Lejman, Jerzy Kowalczyk, Wojciech Fendler, Wojciech Młynarski

**Affiliations:** 1 Department of Pediatrics, Oncology and Hematology, Medical University of Lodz, Lodz, Poland; 2 Department of Pathology, Chair of Oncology, Medical University of Lodz, Lodz, Poland; 3 Department of Pediatric Hematology and Oncology, Medical University of Lublin, Lublin, Poland; 4 Department of Biostatistics & Translational Medicine, Medical University of Lodz, Lodz, Poland; 5 Department of Radiation Oncology, Dana-Farber Cancer Institute, Boston, MA USA

**Keywords:** Childhood BCP-ALL, MLPA, FISH assay, CNVs, Intrachromosomal amplification of chromosome 21 (iAMP21), *ETV6-RUNX1* fusion

## Abstract

Chromosome 21 abnormalities are the most frequent genetic findings in childhood B cell precursor acute lymphoblastic leukemia (BCP-ALL) cases. Majority of patients are effectively diagnosed with fluorescence in situ hybridization (FISH) and karyotyping; however, some cases may require additional tools to be used. Bone marrow samples of 373 childhood BCP-ALL patients were tested for chromosome 21 copy number variations (CNVs) with Multiplex Ligation-dependent Probe Amplification (MLPA) P327 array. Results from MLPA and cytogenetics were compared between groups according to the type of abnormality found on chromosome 21. Out the group of 235 patients, chromosome 21 multiplication was found by FISH assay in 56 cases (23.81%), *ETV6-RUNX1* fusion in 34 (14.47%) and iAMP21 in 3 (1.28%) children, remaining 142 (60.43%) patients had no known chromosome 21 aberration. Median peak ratios of all tested probes in MLPA in aforementioned groups were 1.47 (IQR 1.28–1.77) vs. 1.00 (IQR 1.00–1.09) vs. 2.79 (IQR 1.97–2.83) vs. 1.00 (1.00–1.11), respectively. Aforementioned peak ratio of *ETV6-RUNX1* fusion group was similar with patients of no known chromosome 21 aberration (*p* = 0.71). Interestingly, both groups differed from patients with chromosome 21 multiplication (*p* < 10^−5^) and with iAMP21 (*p* < 10^−5^). All cases of iAMP21 were correctly recognized by MLPA. MLPA seems to be good additional tool in the diagnostic process of chromosome 21 CNVs, especially in cases with iAMP21.

## Introduction

Genetic abnormalities of the chromosome 21 are the most common findings among children diagnosed with B cell precursor acute lymphoblastic leukemia (BCP-ALL) (Li et al. 2014a; Johnson et al. 2015). Molecular subtypes of BCP-ALL with changes in the chromosome 21 are in majority connected with good prognosis, e.g., hyperdiploidy with chromosome 21 multiplication or *ETV6-RUNX1* fusion t(12;21)(p13.2;q22.q) (Depil et al. 1998; Harewood et al. 2003) with 5-year survival rates exceeding 90% in both cases (Brown et al. 2007; Vora et al. 2013, 2014; Gu et al. 2016; Moorman 2016). Recent update of WHO classification for hematologic malignancies defined additional new category of BCP-ALL with aberrations of chromosome 21 that is intrachromosomal amplification of chromosome 21 (iAMP21) (Wenzinger et al. 2018). In contrary to aforementioned changes concerning chromosome 21, iAMP21 is known to be negative predictive and prognostic factor (Heerema et al. 2013; Harrison et al. 2014; Gu et al. 2016; Kim et al. 2016; Yang et al. 2017) if not treated with high-risk protocol.

Those primary genetic abnormalities are identified greatly by karyotyping and fluorescence in situ hybridization (FISH) at the time of diagnosis for further risk stratification and treatment decisions. However, due to low mitotic cells count in the tested sample of bone marrow or low volume of the specimen, up to 30% of ALL patients lack reliable cytogenetic test results (Wang et al. 2016). This revealed the need to fill this gap by alternative diagnostic methods. Among other SNP array, next-generation sequencing or Multiplex Ligation-dependent Probe Amplification (MLPA) seems to be a useful tool for detecting primary genetic aberrations in this subgroup of patients (Harrison et al. 2014; Fuka et al. 2015; Benard-Slagter et al. 2017).

MLPA is a well-known, relatively fast, and efficient method for copy number variation (CNV) detection. On the other hand, several downsides, e.g., semi quantitative results, requirement of high concentration of good quality DNA, cannot be overlooked. A few studies attempted to settle whether MLPA is reliable method in CNVs diagnostics and if it mirrors accurately results from FISH assay; however, conclusions were inconsistent (Garcia et al. 2013; Duployez et al. 2015; Fuka et al. 2015; Ivanov Öfverholm et al. 2016; Wang et al. 2016; Benard-Slagter et al. 2017; Ittel et al. 2017; Yang et al. 2017).

In this study, we have tried to assess the relevance of MLPA as a tool for detecting CNVs at chromosome 21 and compare it with karyotyping and FISH assay results to define its role as complementary tool in diagnostic settings.

## Materials and methods

### Study design and group description

There were 235 children enrolled in the study diagnosed with BCP-ALL between September 2002 and May 2015. The age under 18 years, treatment based on ALL-IC BFM 2002 or 2009, available karyotyping and/or FISH results, and bone marrow sample from the point of diagnosis for DNA extraction were among inclusion criteria. All children were Caucasian, treated in the clinical centers of the Polish Pediatric Leukemia/Lymphoma Study Group. The study was approved by an authorized institutional board and a written consent for participation was required from every patient prior to enrolment.

Out of the collected group, 51.74% (193 patients) were female; median age at the diagnosis was 4.66 (IQR 2.90–8.64). Follow-up time ranged between 0.11 and 12.6 years with mean of 4.31 years. Median blast count of tested samples was 92.40% (84.20–96.00). Patients were divided into four groups according to different chromosome 21 abnormality diagnosed in FISH and karyotyping for further comparisons: chromosome 21 multiplication regardless of hyperdiploidity status, *ETV6-RUNX1* fusion, iAMP21, and cases with no known aberration considering chromosome 21.

### Bone marrow processing

Available samples of bone marrow collected at diagnosis were archived in the TRIzol reagent and stored at – 80 °C. The TRIzol manufacturer’s extraction protocol (Ambion by Life Technologies, Carlsbad, CA, USA) was used. Both DNA quality and quantity were measured at the NanoDrop station (NanoDrop 8000, Thermo Scientific, Waltham, MA, USA).

### Multiplex ligation-depended probe amplification (MLPA)

For all collected bone marrow samples, MLPA analysis with P327 – B1 and B2 iAMP21-ERG probemixes was applied (MRC Holland, Amsterdam, The Netherlands). Data were analyzed using GeneMarker v2.6.3 software according to the manufacturer’s protocol (Softgenetics, State College, PA, USA). The absolute fluorescence was normalized by comparing peak patterns of DNA in the sample of interest with the sample DNA of a healthy individual. The relative probe ratio of tested samples was then compared with average relative probe ratio in the reference samples to calculate Dosage Quotient (DQ). DQ values between 0.85 and 1.15 were considered as normal, between 0.65 and 0.35 as heterozygous deletion, lower than 0.35 as homozygous deletion, between 1.35 and 1.55 as heterozygous duplication, and 1.70 and 2.20 as homozygous duplication. Ratios higher than 2.20 for *RUNX1* probes were considered as iAMP21 amplifications which is higher or similar threshold that was acknowledged in articles considering corresponding analyses (Fuka et al. 2015; Kim et al. 2016).

Data on karyotyping and FISH assay were available for all 235 patients. Tests were conducted by certified external diagnostic laboratory and are basis for both iAMP21 and *ETV6-RUNX1* fusion detection. Hyperdiploid cases with additional chromosome 21 were detected based on FISH method. For iAMP21 confirmation, 5 signals from *RUNX1* specific probe in FISH array must have been detected as a diagnostic criterion (Harrison et al. 2014). MRD at day 15 was measured by flow cytometry in a reference laboratory with 10% being the threshold for a positive result.

### Data analysis

Results with a *p* value lower than 0.05 were considered statistically significant. Statistica 12.0 software (TIBCO Software Inc., Palo Alto, CA, USA) was applied for all computations. Categorical variables are presented as percentages and differences between the groups were calculated with *χ*^2^ test. Continues variables were presented as medians with interquartile range and differences between groups were computed with Kruskal-Wallis test or Mann-Whitney *U* test for paired groups. Post hoc computations were conducted with Tukey’s HSD test. For result presentation, GraphPad Prism 7.05 software (GraphPad Software. La Jolla, CA, USA) was used.

## Results

In all collected cases of BCP-ALL (235 children), cytogenetic testing was used to determine CNVs of genes located on the chromosome 21. Chromosome 21 aberrations were found in 93 (39.57%) bone marrow samples at the time of diagnosis. Among these patients, chromosome 21 multiplication was the most frequent finding (60.22%, 56 cases), *ETV6-RUNX1* fusion was diagnosed in 36.56% (34 cases), iAMP21 amplification in 3.23% (3 children). Forty out of all 60 hyperdiploidic (with more than 51 chromosomes) cases bore chromosome 21 multiplication (66.67%). Described differences in incidence were not statistically significant (Supplementary Table 3). In the group of patients with chromosome 21 gain, the mean count of additional chromosomes 21 was 1.8 with the highest number of 5 additional copies per cell.

### Clinical characteristics of the group

Clinically, groups with chromosome 21 aberrations significantly differed according to the age at diagnosis with iAMP21 positive being the oldest group with median 12.65 years old (*p* = 0.0118). *ETV6-RUNX1* fusion group was predominantly male (58.82%) which is the highest rate among analyzed groups (*p* = 0.5865). Patients without considered primary chromosome 21 abnormalities had the highest WBC at onset of 16.35 × 10^3^/μL (*p* = 0.0286). Median blast count at diagnosis deviated between 84.00 and 94.80% across all groups (*p* = 0.0594). Poor steroid response was most frequent in group with lack of chromosome 21 aberration 11.27% and differences between groups were not significant (*p* = 0.1256). All clinical data are shown in Table 1.

**Table 1:** Clinical characteristics of groups according to the type of chromosome 21 abnormality. *p* values of the group compared to patients with no known chromosome 21 aberration in post hoc Tukey’s HSD test

	No chromosome 21 aberrations (*N* = 142)	Chromosome 21 multiplication (*N* = 56)	*p* value	*ETV6-RUNX1* fusion (*N* = 34)	*p* value	iAMP21 (*N* = 3)	*p* value
Age at diagnosis (year)	4.45 (2.66–8.50)	5.86 (2.86–9.67)	0.43	3.53 (2.80–7.63)	0.95	12.65 (7.18–15.10)	0.81
Female gender (%)	52.82	53.57	1.00	41.18	0.77	66.67	0.99
WBC at diagnosis (× 10^3^/μL)	16.35 (4.85–41.00)	6.47 (3.17–21.40)	0.31	9.17 (4.22–36.88)	0.91	2.30 (1.80–120.00)	1.00
Blast count at diagnosis (%)	90.00 (77.00–95.00)	94.80 (88.00–97.50)	0.23	93.00 (81.00–97.00)	1.00	84.00 (73.00–89.60)	0.99
Risk group (%)	25 (SR, 17.61%)	13 (SR, 23.21%)	0.48	7 (SR, 20.59%)	0.96	0 (SR, 0%)	0.60
	86 (IR, 60.56%)	37 (IR, 66.07%)		21 (IR, 61.76%)		1 (IR, 33.33%)	
	31 (HR, 21.83%)	6 (HR, 10.71%)		6 (HR, 17.65%)		2 (HR, 66.67%)	
CNS involvement at diagnosis (%)	8 (5.63%)	3 (5.36%)	1.00	0 (0%)	1.00	0 (0%)	0.95
Poor steroid response (%)	16 (11.27%)	2 (3.57%)	0.44	1 (2.94%)	0.59	0 (0%)	0.96
MRD at day 15 in flow cytometry (%)	2.45 (0.31–18.15)	0.63 (0.07–4.80)	0.68	0.30 (0.08–3.28)	0.45	27.60 (0.00–52.40)	1.00
5-year OS (%)	89.83 (86.09–93.57)	90.20 (85.07–94.70)	0.96	86.26 (79.67–92.85)	0.90	100%	0.56
5-year RFS (%)	89.20 (86.42–91.98)	80.47 (73.78–87.16)	0.98	83.31 (74.90–91.72)	1.00	66.67%	0.50

Among iAMP21 patients, 1 was treated according to intermediate risk (IR) and 2 as high-risk (HR) group of relapse. For *ETV6-RUNX1* fusion, positive cases and group with chromosome 21 gain only 17.65% and 10.71% of children were treated as HR group, respectively. Among patients with lack of chromosome 21 aberration, 31 cases (21.83%) were stratified to HR treatment protocol.

In case of MRD at day 15, differences between groups were statistically significant (*p* = 0.0114) with median values of 2.45% vs. 0.63% vs. 0.30% vs. 27.60% for group with lack of chromosome 21 aberrations, chromosome 21 gain, *ETV6-RUNX1* fusion, and iAMP21 group, respectively. Analysis of MRD at day 15 positive results in flow cytometry with the cut-off at 10% reported no significant difference between the groups with *p* value of 0.1077, with the highest incidence of positive MRD (66.67% cases) in the iAMP21 group, 19.64% in chromosome 21 gain group, 20.59% in *ETV6-RUNX1* fusion group, and 31.69% in patients with the lack of chromosome 21.

### MLPA results

**Fig. 1:**
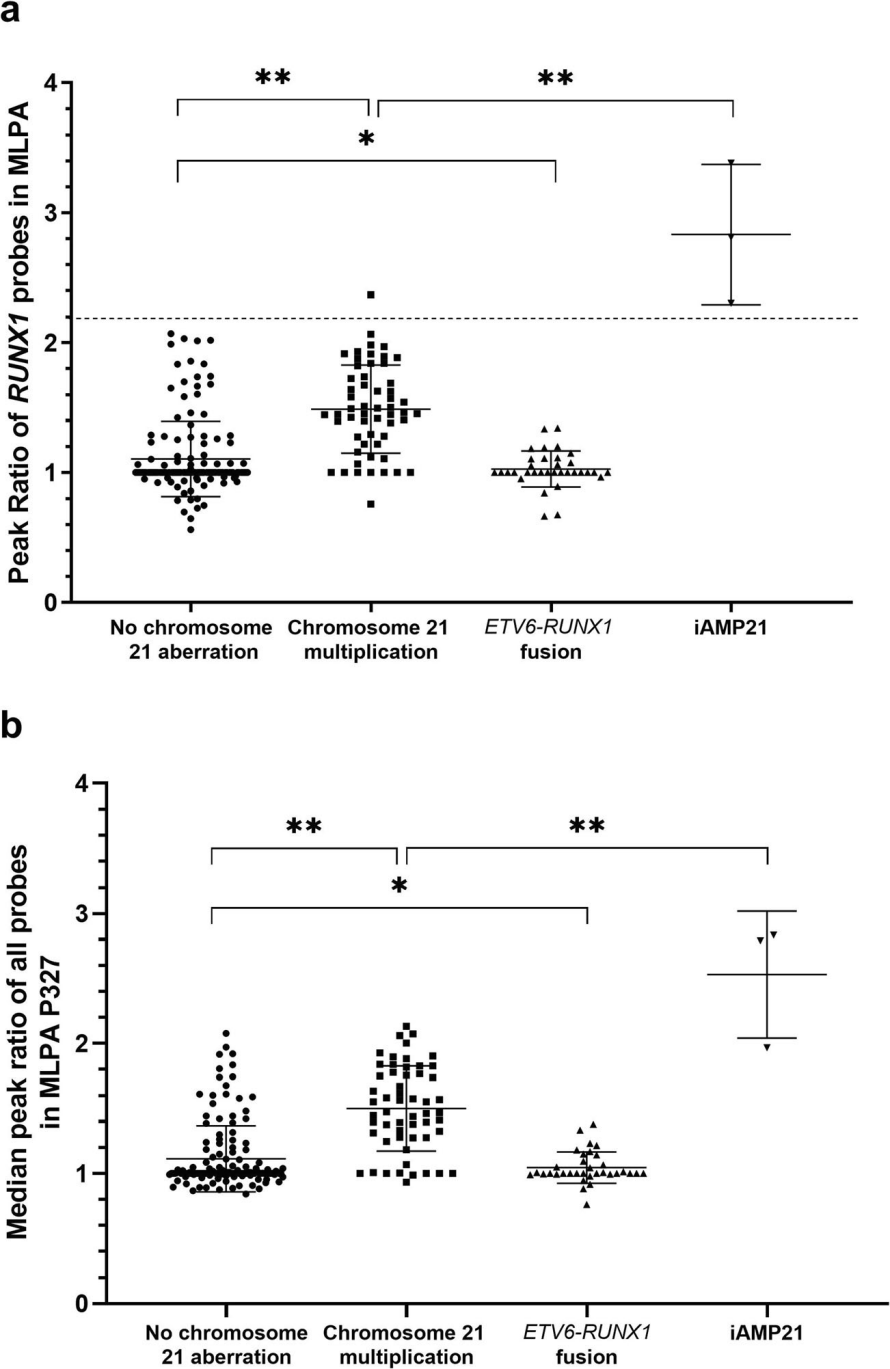
**a** Median peak ratios of *RUNX1* probes in MLPA P327 according to the type of chromosome 21 aberration; *p = 0.6812; **significant difference between median values with *p* < 10^−5^. Dotted line represents threshold of 2.20 above which MLPA suggests iAMP21 diagnosis. **b** Median peak ratios of all probes in MLPA P327 according to the group of chromosome 21 aberration. *p = 0.7094; **statistically significant difference with *p* < 10^−5^

**Fig. 2:**
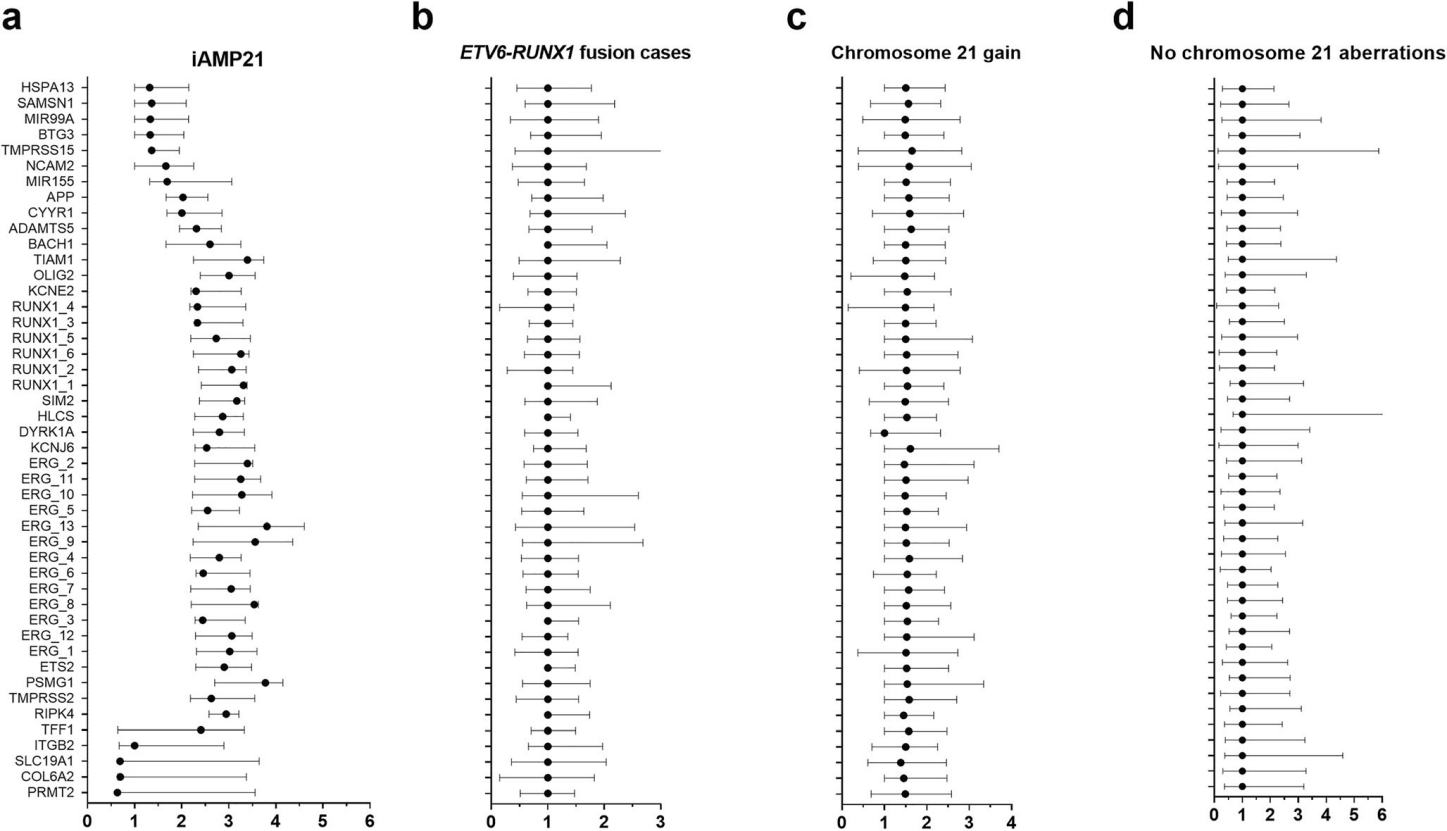
Median values on *x*-axis of each probes from MLPA P327 iAMP21-ERG probemix on *y*-axis in groups. Probes order reflects location on the chromosome 21. Differences of median peak ratios between the groups were statistically significant with *p* < 10^−5^. *ADAMTS5* ADAM Metallopeptidase With Thrombospondin Type 1 Motif 5; *ALL-IC BFM* Acute Lymphoblastic Leukemia Intercontinental Berlin-Frankfurt-Münster; *APP* Amyloid Beta Precursor Protein; *BACH1* BTB Domain And CNC Homolog 1; *BCP-ALL* B cell precursor Acute Lymphoblastic Leukemia; *BTG3* BTG Anti-Proliferation Factor 3; *CISH* Chromogenic in situ hybridization; *CNV* Copy Number Variation; *COL6A2* Collagen Type VI Alpha 2 Chain; *CYYR1* Cysteine And Tyrosine Rich 1; *DYRK1A* Dual Specificity Tyrosine Phosphorylation Regulated Kinase 1A; *ERG* ETS Transcription Factor ERG; *ETS2* ETS Proto-Oncogene 2, Transcription Factor; *FISH* Fluorescent in situ hybridization; *HLCS* Holocarboxylase Synthetase; *HSPA13* Heat Shock Protein Family A (Hsp70) Member 13; *iAMP21* intrachromosomal amplification of chromosome 21; *ITGB2* Integrin Subunit Beta 2; *KCNE2* Potassium Voltage-Gated Channel Subfamily E Regulatory Subunit 2; *KCNJ6* Potassium Voltage-Gated Channel Subfamily J Member 6; *MIR155* MicroRNA 155; *MIR99A* MicroRNA 99a; *MLPA* Multiplex Ligation-dependent Probe Amplification; *MRD* Minimal Residual Disease; *NCAM2* Neural Cell Adhesion Molecule 2; *OLIG2* Oligodendrocyte Transcription Factor 2; *PRMT2* Protein Arginine Methyltransferase 2; *PSMG1* Proteasome Assembly Chaperone 1; *qPCR* quantitative Polymerase Chain Reaction; *RIPK4* Receptor Interacting Serine/Threonine Kinase 4; *RUNX1* RUNX Family Transcription Factor 1; *SAMSN1* SAM Domain, SH3 Domain And Nuclear Localization Signals 1; *SIM2* SIM BHLH Transcription Factor 2; *SLC19A1* Solute Carrier Family 19 Member 1; *SNP* Single Nucleotide Polymorphism; *TFF1* Trefoil Factor 1; *TIAM1* T Cell Lymphoma Invasion And Metastasis 1; *TMPRSS15* Transmembrane Serine Protease 15; *TMPRSS2* Transmembrane Serine Protease 2; *WBC* White blood cell count;

Median peak ratios of all tested MLPA probes varied significantly (*p* < 10^−5^) between distinguished groups and are depicted on the Fig. 1a and Table 2. Median peak ratio for patients with lack of chromosome 21 aberration equaled 1.00 (IQR 1.00–1.11) and was similar to cases with *ETV6-RUNX1* fusion 1.00 (IQR 1.00–1.09) (post hoc *p* = 0.7094). For patients with chromosome 21 gain, median ratio reached 1.47 (IQR 1.28–1.77) that was interpreted as a heterozygous amplification of tested region and the results differed significantly from groups of no known chromosome 21 aberration (post hoc *p* = 0.0001) and *ETV6-RUNX1* fusion (post hoc 0.0001). In cases with iAMP21, peak ratios were the highest with a median value of 2.79 (IQR 1.97–2.83) exceeding values for homozygous duplication in MLPA and differed significantly from aforementioned groups (no known chromosome 21 aberration *p* = 0.0001, chromosome 21 multiplication *p* = 0.0001, ETV6-RUNX1 fusion *p* = 0.0001; all in post hoc analyses). As it is shown on Fig. 1a, there are cases with chromosome 21 gain (10 samples, 17.86%) that have median peak of MLPA P327 probes in the range between 0.85 and 1.15—if basing on these results, they would have been interpreted as normal and are false-negative examples. In the group negative for chromosome 21 CNVs, 22 cases were found to be false positive in MLPA suggesting duplications (15.49%). No other cases of false positive nor negative results were reported. Surprisingly, all groups but iAMP21 are characterized by a unified level of median peak ratios of every probe in applied MLPA probe mixes (Fig. 2).

**Table 2:** Median values and range of peak ratios of all MLPA P327 probes and selectively *RUNX1* probes according to the group. Data from the table are graphically presented by Fig. 2A and B. *p* values represent group comparison with patients of no chromosome 21 aberration

	No chromosome 21 aberrations	Chromosome 21 multiplication	*p* value	*ETV6-RUNX1* fusion	*p* value	iAMP21	*p* value
Median peak ratio of all probes in MLPA P327	1.00 (1.00–1.11)	1.47 (1.28–1.77)	< 0.001	1.00 (1.00–1.09)	0.709	2.79 (1.97–2.83)	< 0.001
Median peak *RUNX1* probes ratio	1.00 (1.00–1.07)	1.46 (1.25–1.73)	< 0.001	1.00 (1.00–1.11)	0.681	2.81 (2.30–3.38)	< 0.001

Subsequently, we analyzed *RUNX1* gene amplification in MLPA to determine iAMP21 cases in collected cohort to assess accuracy of obtained results compared with data from FISH assay. Out of 4 cases with *RUNX1* probes median peak ratio exceeding 2.20 in MLPA, 3 had iAMP21 confirmation. First case of iAMP21 was described with more than 12 signals for *RUNX1* per cell and confirmation in SNP array, the second had confirmed 8–9 copies of *RUNX1* in leukemia clone with karyotype 47,inc/46,XX, and the third 6–9 copies of *RUNX1* per cell and karyotype 46,XY,− 21,+ mar. The fourth patient with *RUNX1* probes peak ratio > 2.20 in MLPA suggesting iAMP21 that was not confirmed by FISH assay was diagnosed with high hyperdiploidy with karyotype 67–68,XXYY,− 1,+ 8,− 9,+ 14,+ 14,− 16,− 19,− 20,+21,+21. In this case, peak ratios of all gene probes, not only *RUNX1* region, at the chromosome 21 in MLPA P327 probemix were unifiably risen that was not typical for iAMP21.

Median peak ratio for all 6 *RUNX1* probes in MLPA of iAMP21 positive cases was 2.81 (IQR 1.97–2.83) and differed from other groups (post hoc *p* = 0.0001 when compared with every group). In contrast, median peak ratio of *RUNX1* probes in *ETV6-RUNX1* fusion cases was within normal limits 1.00 (IQR 1.00–1.11), as well as for samples with no known chromosome 21 aberrations 1.00 (IQR 1.00–1.07) (post hoc *p* = 0.6812). In cases with chromosome 21 multiplication, median *RUNX1* peak ratio reached 1.46 (IQR 1.25–1.73) and was interpreted as heterozygous amplification and differed significantly from aforementioned (post hoc *p* = 0.0001 when compared with every group; Fig. 1b and Table 2). There were no false-negative cases of iAMP21 in MLPA analysis after verification with FISH assay.

### Megabase region of amplification in iAMP21 cases

**Fig. 3:**
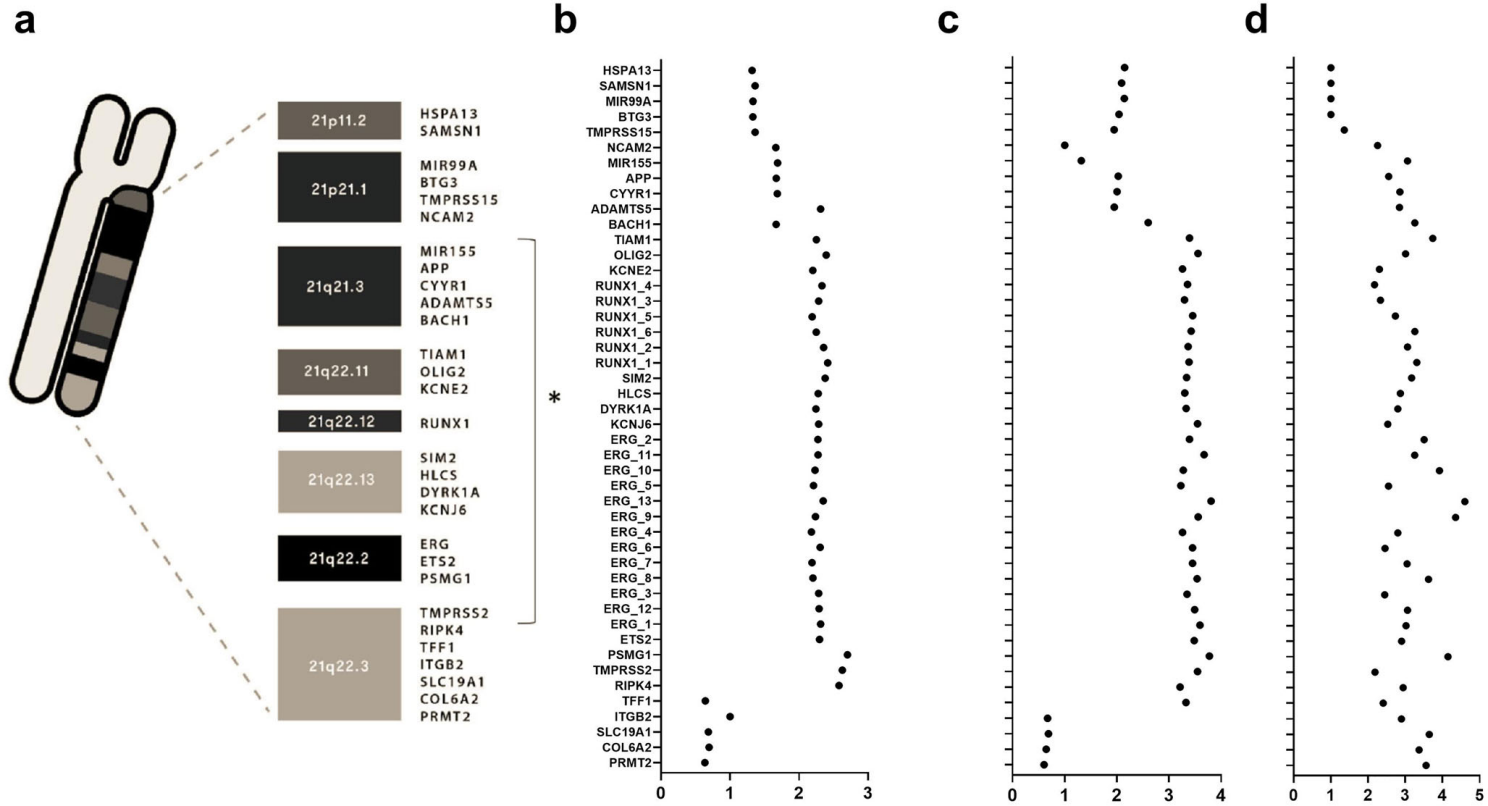
A. Location of tested genes on chromosome 21; *megabase region of amplification characteristic, in this study, only for iAMP21 cases. iAMP21 positive 3 cases (B–D) in MLPA P327 B1 and B2 iAMP21-ERG probemix. Probes lined on *y*-axis according to the location on the chromosome 21. The *x*-axis represents probes’ peak ratios

Interestingly, analysis of all 46 probes in MLPA P327 array for genes located on chromosome 21 between 21q11.2 and 21q22.3 revealed that in cases with confirmed in FISH assay iAMP21, region between genes *NCAM2* (21q21.1) and *RIPK4* (21q22.3) is highly amplified in a megabase block (Fig. 2A and Fig. 3). The size of the common region of amplification region was 20.77 Mbp in average. All cases contained concomitant *ERG* amplification. Surrounding probes between 21q11.2 and 21q21.1 (*HSPA13*, *SAMSN1*, *MIR99A*, *BTG3*, *TMPRSS15*) were characteristically not amplified in all cases of iAMP21 and the region 21q22.3 with genes *TFF1*, *ITGB2*, *SLC19A1*, *COL6A2*, and *PRMT2* was not concomitantly amplified in 2 out of 3 iAMP21 cases. In contrary, cases with variable copy number of the chromosome 21 or *ETV6-RUNX1* fusion have had homogenous level of probes’ peak ratios across MLPA P327 probe mix.

## Discussion

In diagnostic setting for chromosome 21 gain, *ETV6-RUNX1* fusion, and iAMP21, cytogenetics is approved and additionally required to validate any other method used (Harrison et al. 2014). As for CNVs detection in MLPA, among many advantages, like being fast and cost effective method that is of importance in diagnostics, there are major disadvantages to be aware of. For instance, due to blast clone heterogeneity or too low total blast count in tested samples, MLPA might be exposed to high rate of false-negative results since it is based on relative peak ratios and semi quantitative measurement methodology. Reports on MLPA relevance in diagnostics are contradictory. A few publications considering application of MLPA in diagnostic setting of primary aberrations proved inferiority when compared with FISH assay that is undeniably standard method for detection of hyperdiploidy with chromosome 21 gain or *ETV6-RUNX1* fusion, with just several methods, e.g., real-time PCR, karyotyping, and SNP array being appreciated aid (Sinclair et al. 2011; Duployez et al. 2015; Fuka et al. 2015; Kim et al. 2016; Luskin et al. 2017). In contrary, different publications argue that MLPA match results with FISH, CISH array, and qPCR in cases with single-gene CNV detection (Benard-Slagter et al. 2017).

Despite high value of FISH assay and karyotyping, its limitations, as previously mentioned, cannot be overseen and those cases may require alternative diagnostic tools. Surprisingly, in our study, lack of cytogenetic results affected larger group of patients than reported in previous publications (Wang et al. 2016). It was a consequence of missing data in majority of cases, which is a limitation of our study, and rarely low mitotic cell count of the tested sample that limits feasibility of karyotyping. Idealistically, using MLPA as an additional tool for multiple target CNVs assessment would potentiate the process and make it more effective. Being aware of possible false negative and positive cases, MLPA could be cautiously applied in smaller groups of patients that are not suitable for other verification.

Interestingly, in studied group, there were no false-negative results of MLPA array of iAMP21 cases when validated with FISH testing that establishes it as a reliable diagnostic tool in this poor outcome subgroup. Median peak ratios of both *RUNX1* and all probes in MLPA P327 significantly distinguished patients between aforementioned groups, proving consistency of MLPA. Nevertheless, one previously published study of iAMP21 detection in MLPA reported a few false-negative results of iAMP21 detection (Kim et al. 2016).

Unfortunately, MLPA is able to diagnose only copy number variations and will oversee cases of *ETV6-RUNX1* fusion that does not change *ETV6* nor *RUNX1* copy number. As a result, in this study, *ETV6-RUNX1* fusion cases were reported as normal peak ratios across all MLPA P327 probes. Additionally, small number of patients in chromosome 21 gain group had peak ratios in MLPA within normal limits. These false-negative results could be an example of MLPA limitation, due to heterogeneity of the sample with a subclonal chromosome 21 multiplication or low blast count of the sample that is difficult to trace by this semi quantitative method.

Although this study reported iAMP21 positive cases with the megabase block of amplification between *NCAM2* (21q21.1) and *ITGB2* (21q22.3), other amplification regions between *PDE9A* and *COL6A2* were also described (Rand et al. 2011). Previous studies defined molecular basics of intrachromosomal amplification of 21 chromosome that result from telomere attrition initiating breakage-fusion-bridge cycle and consecutive chromothripsis (Li et al. 2014b). As a result genes appear to be more frequently amplified from centromere to telomeric end of the chromosome. This intrachromosomal amplification is said to appear prior even if coexisting with other relevant genetic aberrations (Rand et al. 2011). Surprisingly, reported in this study pattern of megabase amplification in iAMP21 patients is not consistent with the extention of amplification in previous reports (Li et al. 2014a; Tsuchiya et al. 2017). However, the common region of amplification consistently includes *RUNX1*, *DYRK1A*, or *RTS2* with genes behind the region 21q22.3 to be not amplified (Rand et al. 2011; Li et al. 2014b).

Despite limitations, based on the presented study, we can conclude that MLPA is a good tool for diagnosis of iAMP21 and a useful aid in chromosome 21 CNVs detection. Undoubtedly, it could be used as a complementary method for FISH assay in the diagnostic setting.

## Appendix

**Supplementary Table 3:** Molecular characteristics of groups according to the type of chromosome 21 abnormality

	No chromosome 21 aberrations (*N* = 142)	Chromosome 21 multiplication (*N* = 56)	*ETV6-RUNX1* fusion (*N* = 34)	iAMP21 (*N* = 3)	*p* value
Hyperdiploidy (%)	12 (8.45%)	40 (71.43%)	1 (2.94%)	1 (33.33%)	0.0000
Hypodiploidy (%)	8 (5.63%)	0 (0%)	2 (5.88%)	0 (0%)	0.1149
*KMT2A* rearrangements (%)	10 (7.04%)	1 (1.79%)	1 (2.94%)	0 (0%)	0.3381
*BCR-ABL1* fusion (%)	3 (2.11%)	1 (1.79%)	0 (0%)	0 (0%)	0.7042
*IKZF1* del (%)	27 (19.01%)	12 (21.43%)	3 (8.82%)	0 (0%)	0.2603
*TCF3-PBX1* fusion (%)	5 (3.68%)	1 (1.92%)	0 (0%)	0 (0%)	0.6759
